# Cultivar Susceptibility to Natural Infections Caused by Fungal Grapevine Trunk Pathogens in La Mancha Designation of Origin (Spain)

**DOI:** 10.3390/plants10061171

**Published:** 2021-06-09

**Authors:** Juan L. Chacón-Vozmediano, David Gramaje, Maela León, Josep Armengol, Juan Moral, Pedro M. Izquierdo-Cañas, Jesús Martínez-Gascueña

**Affiliations:** 1 Institute for Agri-food and Forestry Research and Development of Castilla-La Mancha (IRIAF), Tomelloso, 13700 Ciudad Real, Spain; pmizquierdo@jccm.es (P.M.I.-C.); jmartinezg@jccm.es (J.M.-G.); 2 Institute of Grapevine and Wine Sciences (ICVV), Spanish National Research Council (CSIC), University of La Rioja and Government of La Rioja, 26007 Logroño, Spain; david.gramaje@icvv.es; 3 Instituto Agroforestal Mediterráneo, Universitat Politècnica de València, 46022 Valencia, Spain; maela.leon@uv.es (M.L.); jarmengo@eaf.upv.es (J.A.); 4 Department of Agronomy, María de Maeztu Unit of Excellence, Campus of Rabanales, University of Córdoba, 14071 Córdoba, Spain; juan.moral@uco.es

**Keywords:** fungal pathogens, grapevine, grapevine trunk diseases, natural infections, pathogenicity

## Abstract

Grapevine trunk diseases (GTDs) are one of the main biotic stress factors affecting this crop. The use of tolerant grapevine cultivars would be an interesting and sustainable alternative strategy to control GTDs. To date, most studies about cultivar susceptibility have been conducted under controlled conditions, and little information is available about tolerance to natural infections caused by GTD fungi. The objectives of this study were: (i) to identify tolerant cultivars to GTD fungi within a Spanish germplasm collection, based on external symptoms observed in the vineyard; and (ii) to characterize the pathogenic mycoflora associated with symptomatic vines. For this purpose, a grapevine germplasm collection including 22 white and 25 red cultivars was monitored along three growing seasons, and their susceptibility for esca foliar symptoms was assessed. Fungi were identified by using morphological and molecular methods. Cultivars such as, ‘Monastrell’, ‘Graciano’, ‘Cabernet Franc’, ‘Cabernet Sauvignon’, ‘Syrah’, ‘Moscatel de Alejandría’, ‘Sauvignon Blanc’, and ‘Airén’ displayed high susceptibility to GTDs, whereas others such as ‘Petit Verdot’, ‘Pinot Noir’, ‘Chardonnay’, and ‘Riesling’ were considered as tolerant. The prevalent fungal species isolated from symptomatic vines were *Phaeomoniella chlamydospora* (27.9% of the fungal isolates), *Cryptovalsa ampelina* (24.6%), and *Dothiorella sarmentorum* (21.3%).

## 1. Introduction

Grapevine trunk diseases (GTDs) are currently considered one of the main types of biotic stress of this crop due to reducing both yield and lifespan of vineyards, which results in substantial economic losses to the grape and wine industry worldwide [1]. GTDs are characterized by presenting a broad diversity of internal wood and foliar symptoms, resulting in an overall decline and eventual death of the affected plants [2]. These diseases are as old as vine cultivation; however, their impact and significance have only been recognized in the early 1990s, when wine growers and the wine industry began to worry about the economic losses that they caused [1]. This emergence is thought to be correlated with several factors, including changes in viticulture practices and vineyard management, and the prohibition of effective fungicides against GTD fungi [1,3,4]. The increasing incidence of GTDs over recent decades is probably related to a sum of pathogen, hostplant, environmental (i.e., abiotic stresses), and cultural factors [5].

The aggressiveness and symptoms caused by fungal pathogens associated with GTDs differ significantly between grapevine-growing regions and vary depending on cultivars [6]. In field trials, one of the main problems to diagnose GTDs is related to the variability in external symptom expression, whereby symptoms on leaves and berries may be obvious one year but are not apparent in another [5]. Furthermore, it is common for several GTDs to overlap in the same grapevine simultaneously [6]. Therefore, it is difficult to associate visual symptoms with causal agents.

A complex of fungal genera and species of taxonomically unrelated—ascomycetous and basidiomycetous—fungi are associated with GTDs [1], which can cause more than one disease [2]. Fungal GTD complex currently includes six main different diseases affecting both grapevine planting material in nurseries, as well as young and mature vineyards. These diseases are: black-foot, Petri and esca diseases, and Botryosphaeria, Eutypa, and Diaporthe diebacks [1,2,5]. Black-foot and Petri disease affect planting material and young vineyards of up to 8-years-old, whereas esca disease, and Botryosphaeria, Eutypa, and Phomopsis diebacks predominantly affect mature grapevines that are more than eight years old. Among these diseases, Botryosphaeria dieback caused by several species in the Botryosphaeriaceae family is the most widespread worldwide [7,8].

GTDs pathogens can be propagated using infected planting material in nurseries [1]. In mature vineyards, infection of grapevines by these fungi primarily occurs through pruning wounds. Air-borne spores are spread by rain splashes, wind, or arthropods, coming in contact with and colonizing exposed wood vessels [9,10,11]. Grapevines have the highest risk of infection during the pruning period because of the high number of wounds made on a single grapevine and the frequency of rain events that occur during that period. Grapevine wounds remain susceptible to infection for several weeks [9,10].

Currently, there are no effective strategies to control GTDs. Thus, the use of tolerant cultivars could be considered an interesting and sustainable alternative strategy to minimize their incidence. This approach would be the least expensive, and the most effective means of controlling them [1]. Phenotyping assays to determine the susceptibility of grapevines to GTDs fungi have mainly focused on two directions: (i) mechanical artificial inoculations of the fungi on plant material—cultivar cuttings or canes—under laboratory, greenhouse, and field conditions [12,13,14,15,16,17,18,19,20,21,22,23,24], and (ii) field observations of natural fungal infections [14,20,25,26,27]. In the latter case, the undetermined latency period (asymptomatic status) and the “erratic” behavior of the foliar symptoms displayed for these diseases, especially the esca complex, make the implementation of long-term studies under field conditions necessary.

La Mancha Designation of Origin (DO) (Central Spain) is the largest delimited viticultural area in Europe (157,449 ha) and one of the most important wine-growing regions in the world. In this work, a vineyard with 47 cultivars authorized in this DO has been monitored for three growing seasons to characterize their susceptibility to GTDs, based on visual assessment of external symptoms observed in grapevines, complemented at the end of the third year with the isolation of fungi. The main objectives were: (i) to identify tolerant cultivars to GTDs fungi in a Spanish germplasm collection, based on external symptoms observed in the vineyard; and (ii) to characterize the pathogenic mycoflora associated with symptomatic vines. In the latter case, problematic aspects related to the indeterminate latency period (asymptomatic state) and the “erratic” behavior of the foliar symptoms that these diseases present, especially the Esca complex, make it necessary for studies to be carried out over several years This is the first study carried out about the susceptibility of grapevine cultivars to GTD infections in the La Mancha region. Knowledge on cultivar resistance to fungal trunk pathogens is critical for growers who plant to establish or replant vineyards and wish to reduce their reliance on fungicides and costs for controlling GTDs. This study will also provide information about the prevalent fungal species associated with GTDs in La Mancha DO.

## 2. Results

### 2.1. GTD Assessment

**Figure 1:**
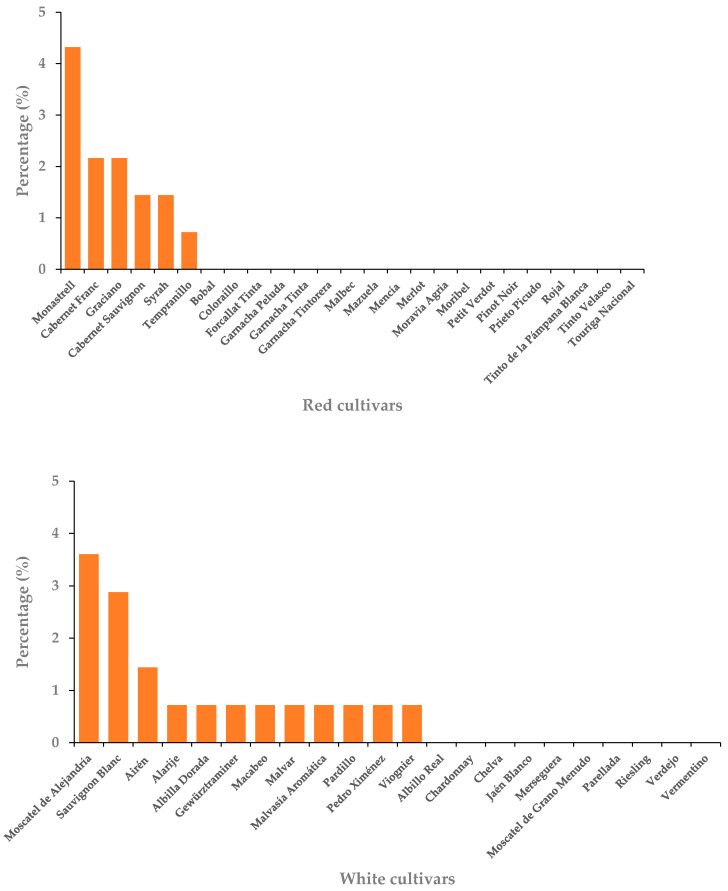
Percentage of grapevines showing foliar/external symptoms associated with GTDs in the different cultivars along with the growing seasons 2016, 2017, and 2018.

Of the 47 cultivars studied, only 18 of them (38.3%) showed symptoms associated with GTDs in at least one vine. The number of vines showing external symptoms was 37 (0.57% of the vines in the vineyard). Figure 1 shows the percentage of symptomatic vines with respect to the total vines of each cultivar (*n* = 139). The highest values corresponded to the cultivars ‘Monastrell’, ‘Moscatel de Alejandría’, and ‘Sauvignon Blanc’ with values of 4.32%, 3.60%, and 2.88%, respectively, on the total vines of each cultivar.

According to the Friedman’s test, both the season and the grapevine cultivar significantly (*p* < 0.05) influenced the severity of GTDs symptoms. Regarding disease severity, the cultivars were grouped into six homogeneous groups with the cultivars ‘Monastrell’ and ‘Moscatel de Alejandría’ being the most susceptible (Table 1). However, when the cultivars were classified considering the presence or absence of the disease (i.e., the disease incidence) during the last evaluation, the cultivars that did have no symptomatic plants (29 cultivars) were classified as more resistant than those cultivars that had at least one replicated plant showing symptoms, which formed a homogeneous group.

### 2.2. Fungal Isolation and Identification

Fungi were mainly isolated from the central part of the wood of shoots and arms, collected from the grapevines showing external symptoms. Based on colony morphology, conidial characteristics, molecular approaches, and phylogenetic analyses, 61 fungal isolates had 99–100% identity with reference isolates of seven species belonging to the genera *Cryptovalsa*, *Diaporthe*, *Diplodia*, *Dothiorella*, *Phaeoacremonium*, *Phaeomoniella*, and *Phellinus* (Table 2 and Table 3) (Supplementary Figure S1). The prevalent species were *Phaeomoniella chlamydospora* (27.9% of the fungal isolates), *Cryptovalsa ampelina* (24.6%), and *Dothiorella sarmentorum* (21.3%). The remaining isolates were identified as *Diplodia seriata* (11.5%), *Phaeoacremonium minimum* (8.2%), *Diaporthe* sp. (4.9%), and *Phellinus mori* (1.6%). Regarding the family, the species belonging to Botryosphaeriaceae—*D. sarmentorum* and *D. seriata*—were the most prevalent fungi isolated from symptomatic vines (32.8%).

Esca was the most prevalent disease observed during the experiment. It was detected in 20 plants (54.1% of the total symptomatic vines), followed by Botryosphaeria, Eutypa, and Phomopsis diebacks with seven (18.9%), six 16.2%), and three (8.1%) affected vines, respectively. There was only one vine (2.7%) of the ‘Tempranillo’ cultivar showing GTD-external symptoms; however, no GTDs-fungi were isolated from this vine.

## 3. Discussion

This is the first study aimed at assessing the cultivar susceptibility to natural infections caused by fungal GTDs pathogens in a grapevine germplasm collection, in a DO in Spain. To date, no evidence of qualitative resistance to any of the most common GTDs fungi has been found. Several infection assays have reported varying GTD resistance of grapevine cultivars to these pathogens [13,17,20,23,24,27], clones [27,28], and rootstocks [29,30,31,32,33], but the vine defense mechanisms underlying those observations, which would explain the tolerance or susceptibility of the different cultivars, have not yet been completely elucidated. Among the different reasons that may cause the difference in susceptibility between cultivars, small xylem vessel diameter and high lignin content in the wood of shoots and arms have been hypothesized to explain tolerance toward fungal vascular pathogens [34,35].

According to GTD symptoms severity observed in our study, six homogeneous groups of cultivars were established. The cultivars in which the symptoms were more severely expressed were coincident with those with the highest number of infected plants. The most severe symptoms were observed in cultivars such as ‘Monastrell’, ‘Moscatel de Alejandría’, ‘Sauvignon Blanc’, ‘Cabernet Franc’, ‘Graciano’, ‘Syrah’, ‘Airén’, and ‘Cabernet Sauvignon’, whereas cultivars ‘Macabeo’, ‘Gewürztraminer’, ‘Alarije’, ‘Pardillo’, ‘Albilla Dorada’, ‘Malvasía Aromática’, ‘Malvar’, ‘Tempranillo’, ‘Pedro Ximénez’, and ‘Viognier’ showed less severe GTDs symptomatology. The remaining 29 cultivars did not show any symptoms.

Previous reports on cultivar susceptibility to esca disease displayed varying results depending on whether the infection occurred artificially or naturally, and also on the environment (in vitro, greenhouse or field) in which the assays were carried out. In studies performed by artificial inoculation of GTDs pathogens, ‘Cabernet Sauvignon’ was shown to be a highly tolerant genotype to *Pa. chlamydospora* in assays performed in vitro [16], and to *Pa. chlamydospora* and *Pm. minimun* in greenhouse [17] and field [12] conditions. In contrast, ‘Cabernet Sauvignon’ was considered a susceptible cultivar to esca natural infection under field conditions in Italy [27,36,37,38], and Australia [20], which is in agreement with the results of our study. ‘Tempranillo’ cultivar has also been widely evaluated to esca disease susceptibility. In contrast with our results, ‘Tempranillo’ was considered as susceptible to *Pa. chlamydospora* infection in assays performed in Portugal [22] and Spain [23] in greenhouse conditions, and to GTD natural infections under field conditions [20,37]. Although cultivar Tempranillo showed GTD external symptoms, no GTD fungi were isolated from this cultivar. This could be due to the sampling methodology and the selection of specific pieces of wood for fungal isolation. In our study, ‘Sauvignon Blanc’ and ‘Syrah’ were considered as susceptible cultivars to esca disease, whereas ‘Merlot’, ‘Chardonnay’, and ‘Riesling’ were considered as tolerant. These findings are in agreement with those obtained by other authors when evaluating GTD natural infections under field conditions, who also considered ‘Sauvignon Blanc’ as susceptible [27,38] and ‘Merlot’ [26,38], and ‘Chardonnay’ [27] as tolerant cultivars. By contrast, other researchers considered ‘Syrah’ and ‘Riesling’ as tolerant and susceptible cultivars, respectively, to GTDs natural infections [27].

Regarding Botryosphaeria dieback, several inconsistences were found between the results of our study and other research carried out worldwide. For example, in a previous study, the severity of internal wood symptoms caused by *Neofusicoccum parvum* differed amongst several cultivars belonging to the germplasm collection evaluated here, being ‘Monastrell’ one of the most tolerant cultivars [24]. In vitro studies showed that ‘Cabernet Sauvignon’ was tolerant to artificial inoculation by *D. seriata*, while ‘Gewürztraminer’ was considered susceptible [14,19]. Further research under field conditions reported a high tolerance of ‘Cabernet Sauvignon’ to Botryosphaeria dieback natural infection, whereas ‘Syrah’ was considered susceptible to this disease [14], which disagrees with the results of the present study. Conversely, in assays performed in field conditions, ‘Syrah’ and ‘Sauvignon Blanc’ were considered as susceptible cultivars, whereas ‘Gewürztraminer’ was considered as moderately susceptible to artificial inoculation by *D. seriata* [20], which partially agree with our results.

In this study, ‘Petit Verdot’, ‘Merlot’, ‘Tempranillo’, ‘Chardonnay’, and ‘Gewürztraminer’ displayed more tolerance to infection caused by *C. ampelina* than ‘Graciano’, ‘Monastrell’, ‘Syrah’, ‘Cabernet Franc’, and ‘Sauvignon Blanc’. These results are generally consistent with those obtained earlier by other researchers [14,20,26], who assessed the tolerance of several cultivars to natural GTD infection in field conditions, and considered ‘Merlot’, ‘Petit Verdot’, and ‘Gewürztraminer’ among the most tolerant cultivars to Eutypa dieback, whereas ‘Chardonnay’, ‘Tempranillo’, and ‘Sauvignon Blanc’ were considered as susceptible cultivars.

Regarding the tolerance to the genus *Phellinus*, in a study performed by artificial inoculation in greenhouse conditions, ‘Cabernet Sauvignon’ and ‘Merlot’ performed as genotypes more tolerant to *Phellinus* sp. and *Ph. punctatus* than ‘Garnacha’ [13].

In studies performed by artificial inoculation in greenhouse, ‘Cabernet Sauvignon’ and ‘Merlot’ performed as more tolerant cultivars to *D. ampelina* than ‘Cabernet Franc’, ‘Chardonnay’, and ‘Riesling’ [17]. These results are consistent with those obtained in this study, in which the only cultivars showing susceptibility to *Diaporthe* sp. were ‘Sauvignon Blanc’ and ‘Gewürztraminer’, whereas the remaining cultivars such as, ‘Cabernet Sauvignon’, ‘Merlot’ ‘Cabernet Franc’, ‘Chardonnay’, and ‘Riesling’ performed as tolerant.

Cultivar susceptibility based on visual assessment of external symptoms [39], mainly foliar symptomatology associated with esca disease [25,26,27,40], has the limitation that the GTD pathogens often occur in mixed infections within the same vine [1]. In contrast with previous studies on natural GTD infection assessment, symptomatic plants were inspected for GTD fungal incidence at the end of the experiment. Isolations from symptomatic vines revealed several pathogens associated with esca disease (*Pa. chlamydospora*, *Pm. minimum*), Eutypa dieback (*C. ampelina*), Botryosphaeria dieback (*D. seriata*, *D. sarmentorum*) and Phomopsis dieback (*Diaporthe* sp.), being *Pa. chlamydospora* the most frequent fungal species, followed by *C. ampelina* and *D. sarmentorum*. In general, these results are consistent with those obtained by other authors in Italy [3] and Spain [41], in which *Pa. chlamydospora* is considered a prevalent pathogen on GTDs symptomatic vines.

*Cryptovalsa ampelina* was previously reported in several regions of Spain [42,43]. This pathogen is mainly found on pruning debris and rarely on standing vines showing symptoms of trunk diseases [42].

The basidiomycete species *Ph. mori* was only isolated in one vine of the ‘Monastrell’ cultivar together with the esca pathogen *Pa. chlamydospora*. This species has not been reported so far as a pathogen associated with GTDs worldwide. The genus *Phellinus* appears to be associated with a secondary stage of the esca disease, colonizing grapevines initially infected by *Pa. chlamydospora* and *Pm. minimum*, which are more prevalent and virulent species [5].

The *Diaporthe* species have been associated with several major diseases of grapevines, such as Phomopsis cane and leaf spot and Diaporthe dieback [44,45,46,47]. The most frequent species isolated of this genus in Europe are *D. eres* and *D. ampelina* (syn. *Phomopsis viticola*) [48]. Both species are shown to be pathogenic on grapevine [48]. Recently, two new *Diaporthe* species were isolated from symptomatic vines collected in Spain, namely *Diaporthe hispaniae* and *Diaporthe hungariae*. These species were closely related, but clearly separated based on morphological and molecular characteristics from *D. ampelina*, historically known as the most virulent *Diaporthe* species of grapevine [47].

The use of tolerant cultivars would be an interesting and sustainable alternative strategy to control GTD infections. This study allowed for classifying several grapevine cultivars according to external symptoms associated with natural infections caused by fungal grapevine trunk pathogens in La Mancha DO, as well as to characterize the pathogenic microflora associated with symptomatic vines in this area. Knowledge of tolerant cultivars to fungal trunk pathogens may help growers to reduce their reliance on fungicides and costs for controlling GTDs. Further research is needed to evaluate the correlation between foliar symptoms and wood deterioration, and to explore the mycoflora associated with asymptomatic vines.

## 4. Material and Methods

### 4.1. Study Area

A plot of the Instituto Regional de Investigación y Desarrollo Agroalimentario y Forestal (IRIAF) was planted with a grapevine germplasm collection and located at 663 m.a.s.l. (latitude: 39.176753N, longitude: −3.000247W) was used. This plot includes a broad range of *V. vinifera* cultivars authorized in the Castilla–La Mancha wine region, which can be considered representative of the vineyards in La Mancha DO. The soil is classified as Calcixerept petric. Its main features are: shallow (<40 cm depth), well-drained, with about 40% of coarse elements and loam to sandy-clay-loam textures (46.6% sand; 32.2% silt, 2.12% clay) and 3.2% of organic matter content [49]. The region has a temperate climate with high differences in temperature between winter and summer. According to the Winkler index, this region is classified as Region IV, and it records scarce rainfall during the year (about 350 mm), with less than 50% occurring in the vine growing season (between vine sprouting and harvesting). The reference evapotranspiration value (ET_0_) is about 1300 mm/year, exceeding 1000 mm during the active vegetation period.

### 4.2. Plant Material

The germplasm collection consists of 22 white (‘Airén’, ‘Alarije’, ‘Albilla Dorada’, ‘Albillo Real’, ‘Chardonnay’, ‘Chelva’, ‘Gewürztraminer’, ‘Jaén Blanco’, ‘Macabeo’, ‘Malvar’, ‘Malvasía Aromática’, ‘Merseguera’, ‘Moscatel de Alejandría’, ‘Moscatel de Grano Menudo’, ‘Pardillo’, ‘Parellada’, ‘Pedro Ximénez’, ‘Riesling’, ‘Sauvignon Blanc’, ‘Verdejo’, ‘Vermentino’, and ‘Viognier’), and 25 red (‘Bobal’, ‘Cabernet Franc’, ‘Cabernet Sauvignon’, ‘Coloraillo’, ‘Forcallat Tinta’, ‘Garnacha Peluda’, ‘Garnacha Tinta’, ‘Garnacha Tintorera’, ‘Graciano’, ‘Malbec’, ‘Mazuela’, ‘Mencía’, ‘Merlot’, ‘Monastrell’, ‘Moravia Agria’, ‘Moribel’, ‘Petit Verdot’, ‘Pinot Noir’, ‘Prieto Picudo’, ‘Rojal’, ‘Syrah’, ‘Tempranillo’, ‘Tinto de la Pámpana Blanca’, ‘Tinto Velasco’, and ‘Touriga Nacional’) grapevine cultivars grafted onto 110 Richter rootstock and planted in 2002 with one panel of 139 vines per cultivar. The information used in this research referred to 47 cultivars with 139 grapevines each (6533 vines in total). The planting pattern was 3 m between rows and 1.5 m between plants (density of 2222 grapevines/ha). All vines were double cordon trained and spur pruned, with no specific strategies to control GTDs. Vines were cultivated under irrigated conditions by a drip system with two drippers per grapevine. Irrigation was applied considering about 25% of crop evapotranspiration, and it represented 120–150 mm per year, on average. The rows were positioned 120°E-SE/300°W-NW.

### 4.3. GTD Assessment and Fungal Isolations

All cultivars were inspected four to five times per season during the vegetative period during the growing seasons 2016, 2017, and 2018, between flowering and maturity, which is the time when GTDs symptoms are most evident. The cultivar susceptibility was assessed for esca foliar symptoms according to a scale ranging from 0 to 5, depending on the affectation level and GTD-associated cordon dieback (partially or totally dry) [50] (Table 4).

Vines showing either esca foliar or cordon dieback symptoms were marked and recorded. GTD fungal isolations were performed from these vines at the end of the study (2018) to correlate foliar symptoms and fungal incidence. In total, 37 samples were collected from 18 different cultivars.

Fungal isolations were carried out according to the methodology described by [51]. Pieces of wood from symptomatic canes and arms were debarked and cut into transverse slices approximately 1 mm thick. These slices were then surface disinfected by immersion in 70% alcohol for 1 or 2 min, depending on thickness, and air dried on sterile filter paper. Later, they were placed in plates of malt extract agar supplemented with 0.5 g/L of streptomycin sulfate (MEAS) and incubated at 25 °C in darkness for 10 days. The plates were observed daily to check the growth of the mycelium. Fungal colonies were transferred to new Petri dishes with Potato Dextrose Agar (PDA) and incubated at 25 °C in darkness, to obtain pure cultures. From these primary isolations, single spore or hyphal tipped isolates were obtained previously to their identification.

### 4.4. Morphological and Molecular Identification of Fungal Cultures

Preliminary morphological identification of the isolates at different taxonomical levels was carried out by observing the cultural and microscope characters of the colonies under stereoscope and microscope, respectively [52]. Colonies were then tentatively grouped as Basidiomycetes, or fungi belonging to the families Botryosphaeriaceae, and Diatrypaceae, the genus *Phaeoacremonium*, and the species *Pa. chlamydospora*.

For species identity confirmation, total fungal DNA was extracted from fungal cultures grown on PDA medium, using the E.Z.N.A. Plant DNA Kit (Omega Bio-tek, Norcross, GA, USA), following the manufacturer’s instructions. Diatrypaceae, Basidiomycetes, and *Pa. chlamydospora* isolates were identified based on the sequence of the ITS region, Botryosphaeriaceae isolates were identified based on the sequences of ITS and a portion of translation elongation factor 1-alpha (tef-1α) region, while, for *Phaeoacremonium* isolates, part of the β-tubulin gene (tub) was used. The primer pairs used for amplification and sequencing of each region were as follows: ITS1-F [53] and ITS4 [54] for ITS, EF1-688F and EF1-1251R for tef-1α [55] and BtCadF and BtCadR for tub [56]. Amplification by polymerase chain reaction (PCR) was performed in a total volume of 25 µL using HotBegan™ Taq DNA Polymerase (Canvax Biotech SL, Córdoba, Spain), according to the manufacturer’s instructions on a Peltier Thermal Cycler-200 (MJ Research). One reaction was composed of 1× PCR Buffer B, 2.5 mM of MgCl2, 0.8 mM of dNTPs, 0.4 µM of each primer, 1 U of HotBegan Taq DNA Polymerase, and 1 µL of purified template DNA. The PCR cycling conditions consisted of an initial step of 3 min at 94 °C, followed by 35 cycles of denaturation at 94 °C for 30 s, annealing at 55 °C for 30 s and elongation at 72 °C for 45 s. A final extension was performed at 72 °C for 5 min. PCR products were confirmed by 1.2% agarose gel electrophoresis and were purified and sequenced by Macrogen Inc. (Madrid, Spain) using both PCR primers. Sequences were assembled and edited using Sequencher software 5.0 (Gene Codes Corp., Ann Arbor, MI, USA). The isolate identities were based mainly on BLASTn searches in NCBI, but, for Botryosphaeriaceae isolates, the multiple sequence alignments and maximum likelihood phylogenetic analyses were conducted in MEGA X [57], using closely related ex-type or representative species as phylogenetic reference.

### 4.5. Data Analysis

Mean, standard deviation, and sum were calculated using the descriptive process of the software Statistix 10 (Analytical Software; Tallahassee, FL, USA). The effect of the evaluation season and the grapevine cultivar on the disease severity were examined using Friedman’s test. For that, the back-transformation of the rating scale was used. Friedman’s test was used because the dependent variable does not satisfy the requirements of parametric tests. The means were compared using Dunn’s test with a Bonferroni adjustment after a Kruskall–Wallis test at *p* = 0.05 [58]. A Zar’s test of multiple comparisons of proportions was performed to study the effect of the cultivar on disease presence (1) or absence (0) in the last studied season (2018) [59]. Data were analyzed using the software Statistix 10 (Analytical Software; Tallahassee, FL, USA) and SPSS (version 19; SPSS Inc., Chicago, IL, USA).

## Figures and Tables

**Table 1:** Homogeneous groups of grapevine cultivars according to severity of grapevine trunk diseases (GTDs) symptoms showed under field conditions in La Mancha Designation of Origin (DO), Central Spain.

Group	n	Cultivars	Mean Rank	Homogeneous Group
1	2	‘Monastrell’	10,122.03 10,097.48	A
		‘Moscatel de Alejandría’		
2	1	‘Sauvignon Blanc’	10,028.47	AB
3	2	‘Cabernet Franc’	9958.05 9933.76	ABC
		‘Graciano’		
		‘Syrah’	9887.52	BCD
4	3	‘Airén’	9887.01	
		‘Cabernet Sauvignon’	9887.01	
5		‘Macabeo’	9816.83	CD
		‘Gewürztraminer’	9816.69	
		‘Alarije’	9816.69	
		‘Pardillo’	9816.69	
	10	‘Albilla Dorada’	9816.44	
		‘Malvasía Aromática’	9816.44	
		‘Malvar’	9816.31	
		‘Tempranillo’	9816.26	
		‘Pedro Ximénez’	9816.16	
		‘Viognier’	9816.16	
6		‘Albillo Real’	9746.00	D
		‘Bobal’	9746.00	
		‘Chardonnay’	9746.00	
		‘Chelva’	9746.00	
		‘Coloraillo’	9746.00	
		‘Forcallat Tinta’	9746.00	
		‘Garnacha Peluda’	9746.00	
		‘Garnacha Tinta’	9746.00	
		‘Garnacha Tintorera’	9746.00	
		‘Jaén Blanco’	9746.00	
		‘Malbec’	9746.00	
		’Mazuela’	9746.00	
		‘Mencía’	9746.00	
		’Merlot’	9746.00	
	29	‘Merseguera’	9746.00	
		‘Moravia Agria’	9746.00	
		‘Moribel’	9746.00	
		‘Moscatel de Grano Menudo’	9746.00	
		‘Parellada’	9746.00	
		‘Petit Verdot’	9746.00	
		‘Pinot Noir’	9746.00	
		‘Prieto Picudo’	9746.00	
		‘Riesling’	9746.00	
		‘Rojal’	9746.00	
		‘Tinto de la Pámpana Blanca’	9746.00	
		‘Tinto Velasco’	9746.00	
		‘Touriga Nacional’	9746.00	
		‘Verdejo’	9746.00	
		‘Vermentino’	9746.00	

Significant differences according to Friedman’s test at *p* = 0.05. Homogeneous. groups were formed according to Dunn’s test corrected by Bonferroni.

**Table 2:** Fungal trunk pathogens isolated from red cultivars showing foliar/external symptoms in 2018.

Cultivar	IdentificationNumber	Foliar/External Symptom Incidence	Fungal Species	Trunk Disease
‘Cabernet Franc’	95		*Pa. chlamydospora*	Esca
		3	*C. ampelina*	
			*D. sarmentorum*	
	101	5	*D. sarmentorum*	Botryosphaeria dieback
	123	5	*D. sarmentorum*	Botryosphaeria dieback
‘Cabernet Sauvignon’	75	3	*C. ampelina*	Eutypa dieback
			*D. seriata*	
	101	3	*D. seriata*	Botryosphaeria dieback
‘Graciano’	12	1	*Pa. chlamydospora*	Esca
			*C. ampelina*	
			*D. sarmentorum*	
	21	5	*C. ampelina*	Eutypa dieback
	84	1	*C. ampelina*	Eutypa dieback
‘Monastrell’	19	3	*Pa. chlamydospora*	Esca
	79	3	*Pa. chlamydospora*	Esca
			*Ph. mori*	
	87	3	*Pa. chlamydospora*	Esca
			*C. ampelina*	
	105	3	*Pa. chlamydospora*	Esca
			*Pm. minimum*	
	109	3	*Pm. minimum*	Esca
			*D. sarmentorum*	
	116	4	*Pa. chlamydospora*	Esca
			*Pm. minimum*	
			*C. ampelina*	
‘Syrah’	39	4	*Pa. chlamydospora*	Esca
			*C. ampelina*	
	51	5	*Pa. chlamydospora*	Esca
			*D. sarmentorum*	
			*D. seriata*	
‘Tempranillo’	35	2	None	Not detected

**Table 3:** Fungal trunk pathogens isolated from white cultivars showing foliar/external symptoms in 2018.

Cultivar	IdentificationNumber	Foliar/External Symptom Incidence	Fungal Species	Trunk Disease
‘Airén’	16	3	*Pa. chlamydospora*	Esca
			*C. ampelina*	
			*D. sarmentorum*	
	79	3	*D. sarmentorum*	Botryosphaeria dieback
‘Alarije’	126	4	*Pa. chlamydospora*	Esca
			*C. ampelina*	
‘Albilla Dorada’	36	3	*C. ampelina*	Eutypa dieback
			*D. seriata*	
‘Gewürztraminer’	22	4	*Diaporthe* sp.	Phomopsis dieback
‘Macabeo’	42	5	*D. sarmentorum*	Botryosphaeria dieback
			*D. seriata*	
‘Malvar’	101	2	*Pa. chlamydospora*	Esca
			*D. sarmentorum*	
‘Malvasía Aromática’	13	3	*D. seriata*	Botryosphaeria dieback
‘Moscatel de Alejandría’	45	2	*Pa. chlamydospora*	Esca
	46	2	*Pa. chlamydospora*	Esca
	47	2	*Pa. chlamydospora*	Esca
			*C. ampelina*	
	48	2	*Pa. chlamydospora*	Esca
			*D. sarmentorum*	
	50	2	*Pm. minimum*	Esca
			*D. sarmentorum*	
‘Pardillo’	96	4	*C. ampelina*	Eutypa dieback
‘Pedro Ximénez’	91	1	*Pm. minimum*	Esca
‘Sauvignon Blanc’	37	5	*Diaporthe* sp.	Phomopsis dieback
	89	4	*D. sarmentorum*	Botryosphaeria dieback
			*D. seriata*	
	92	2	*Diaporthe* sp.	Phomopsis dieback
	107	4	*Pa. chlamydospora*	Esca
			*C. ampelina*	
‘Viognier’	96	1	*C. ampelina*	Eutypa dieback

**Table 4:** Scale of foliar/external symptoms incidence according to percentage of affected vegetation.

Level	Foliar/External Symptom Incidence (%)
0	0
1	0–10
2	11–25
3	26–50
4	51–80
5	>80

## Supplementary Materials

The following are available online at https://www.mdpi.com/article/10.3390/plants10061171/s1, Figure S1: Maximum likelihood phylogeny inferred from the alignment of combined sequences of a portion of translation elongation factor 1-alpha (tef-1α) region and internal transcribed spacers (ITS). Support values higher than 70% are given at the nodes. The tree was rooted using *Neofusicoccum luteum* (CBS110299 and CBS140738) as outgroup sequences. Scale bar shows expected changes per site. Species isolated in this study are indicated in bold. This analysis was conducted in MEGAX, and involved 26 nucleotide sequences. There were a total of 825 positions in the final dataset (EF:1-313 and ITS:314-825).
