# Supplementary material for: Cultivar Susceptibility to Natural Infections Caused by Fungal Grapevine Trunk Pathogens in La Mancha Designation of Origin (Spain)
Source: Plants (Basel). 2021 Jun 9;10(6):1171. doi: 10.3390/plants10061171 (PMC8228040; doi:10.3390/plants10061171)
Supplement: Supplementary file 1 [file plants-10-01171-s001.zip › plants-1199956-supplementary.pdf]

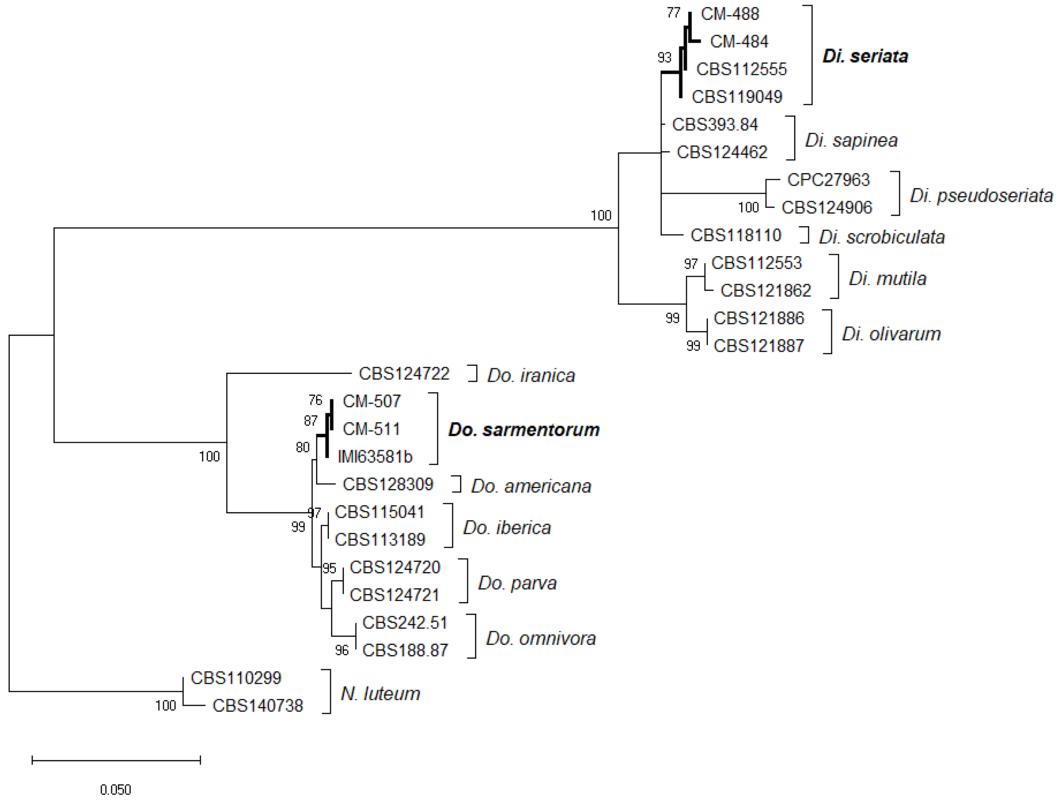

Maximum likelihood phylogeny inferred from the alignment of combined sequences of a portion of translation elongation factor 1-alpha (tef-1α) region and internal transcribed spacers (ITS). Support values higher than 70% are given at the nodes. The tree was rooted using *Neofusicoccum luteum* (CBS110299 and CBS140738) as outgroup sequences. Scale bar shows expected changes per site. Species isolated in this study are indicated in bold. This analysis was conducted in MEGAX, and involved 26 nucleotide sequences. There were a total of 825 positions in the final dataset (EF:1-313 and ITS:314-825).
